# Integrated Transcriptomics and Lipidomics Identify CES1-Mediated Maladaptive Lipolysis as a Key Target of Hyperlipidemic Acute Pancreatitis

**DOI:** 10.3390/metabo16090644

**Published:** 2026-09-03

**Authors:** Jiayu Liu, Yunshu Zhang, Xingchi Jiang, Haocheng Xue, Peiyuan Yin

**Affiliations:** 1 Department of General Surgery, The First Affiliated Hospital of Dalian Medical University, Dalian 116000, China; 2 Institute of Integrative Medicine, Dalian Medical University, Dalian 116000, China; 3 Laboratory of Integrative Medicine, The First Affiliated Hospital of Dalian Medical University, Dalian 116000, China

**Keywords:** hyperlipidemic acute pancreatitis, lipid metabolism, lipidomics, CES1, WWL113

## Abstract

Background: Hyperlipidemic acute pancreatitis (HAP) is a severe disease driven by systemic lipid overload. While free fatty acids (FFAs) are known to mediate pancreatic lipotoxicity, the intracellular enzymatic mechanisms generating these toxic lipid mediators remain unclear. We aimed to identify the core metabolic drivers linking systemic hyperlipidemia to local pancreatic injury and evaluate targeted prophylactic strategies for HAP. Methods: We integrated public transcriptomic datasets of severe AP and obesity/hyperlipidemia. Three machine learning algorithms were employed to identify comorbidity-associated signature genes. The underlying mechanisms were explored via gene set variation analysis, immune infiltration profiling, and single-cell in silico knockout. In vivo validation was performed using a P-407/caerulein-induced HAP mouse model treated with WWL113, followed by comprehensive histological, biochemical, and lipidomic analyses. Results: A robust three-gene signature (FASN, CES1, IL10) was identified with excellent diagnostic accuracy. Notably, within this signature, the triglyceride hydrolase CES1 was aberrantly upregulated, serving as the primary driver of a maladaptive lipolytic shift. CES1 overexpression was strongly correlated with neutrophil infiltration. Single-cell virtual knockout suggested a potential association between Ces1d and markers of endothelial barrier disruption and neutrophil chemotaxis. In vivo, WWL113 significantly attenuated HAP-induced pancreatic necrosis and systemic inflammation. Crucially, lipidomics confirmed that WWL113 sequestered exogenous lipids in inert triglyceride states, drastically reducing toxic FFAs. Conclusions: This study highlights CES1 as a critical intracellular mediator of lipotoxicity in HAP. Pharmacological inhibition of CES1 effectively halts maladaptive lipolysis, providing proof-of-mechanism for a metabolism-directed prophylactic strategy for HAP.

## 1. Introduction

Hyperlipidemic acute pancreatitis (HAP) is a severe clinical subtype characterized by rapid disease progression and a high rate of complications [1]. Epidemiological evidence indicates that obesity and systemic hyperlipidemia are major risk factors that exacerbate acute pancreatitis (AP) into severe disease [2]. In the pathophysiological progression of HAP, systemic lipid metabolic burden intertwines with acute inflammatory cascades in local tissues. Current evidence suggests that circulating triglycerides (TGs) are relatively inert, whereas free fatty acids (FFAs) generated by excessive TG hydrolysis are the principal mediators of lipotoxicity, inducing mitochondrial dysfunction and membrane damage [3,4]. Despite the well-documented role of lipotoxicity, the intracellular enzymatic mechanisms driving pathological TG hydrolysis within the pancreas remain largely undefined.

Lipid metabolic reprogramming and immune dysregulation are closely associated with the progression of HAP [5,6]. Due to the scarcity of clinical transcriptomic cohorts specifically focusing on HAP, we adopted an exploratory, cross-phenotype intersection strategy. By integrating transcriptomic datasets of obesity/hyperlipidemia and severe acute pancreatitis (SAP), we aimed to generate hypotheses regarding metabolic vulnerability factors that may predispose hyperlipidemic individuals to exacerbated pancreatic injury. We emphasize that this approach is hypothesis-generating rather than directly profiling clinical HAP; definitive mechanistic validation was subsequently reserved for our in vivo HAP models. In HAP, lipotoxicity is not limited to direct acinar cell death [7]. The accumulation of toxic lipid intermediates also serves as a danger signal, promoting excessive infiltration and activation of innate immune cells, particularly neutrophils and macrophages [8,9]. Understanding how these metabolic genes orchestrate lipotoxicity and immune responses is crucial for discovering novel therapeutic targets.

In this study, we sought to identify key metabolic mediators linking systemic hyperlipidemia to pancreatic lipotoxicity in HAP. Through an exploratory cross-phenotype transcriptomic strategy, we prioritized a candidate lipolytic enzyme and subsequently evaluated its pathogenic role and therapeutic potential using the HAP mouse model combined with pharmacological inhibition and metabolic profiling. Our findings support CES1-mediated maladaptive lipolysis as a critical driver of HAP and support its targeting as a prophylactic metabolic strategy.

## 2. Methods

### 2.1. Data Acquisition and Preprocessing

The GSE194331 dataset [10] (57 mild, 20 moderately severe, 10 severe acute pancreatitis, and 32 normal samples) and the GSE59034 dataset [11] (16 baseline pre-bariatric surgery obesity samples and 16 normal samples) were obtained from the GEO database (accessed on 24 January 2026). The GSE59034 dataset was derived from a published clinical cohort, in which plasma TGs were significantly elevated in obese individuals [11]. A comprehensive list of 1765 LMRGs was obtained from a recently published study [12]. The complete list of these LMRGs utilized in our study is provided in Supplementary Table S1.

### 2.2. Identification of Comorbidity DEGs and Functional Enrichment

To account for different analytical platforms, distinct preprocessing pipelines were applied. The GSE59034 was normalized using the Robust Multichip Average algorithm. The GSE194331 underwent low-count filtering, followed by voom transformation to correctly model the mean-variance relationship. Differentially expressed genes (DEGs) were identified using the “limma” package (version 3.50.3) [13], with the significance thresholds set at |log2 Fold Change (FC)| > 0.585 and an adjusted *p*-value < 0.05 [14]. The overlapping comorbidity DEGs between the two datasets were identified using a Venn diagram. Functional enrichment analyses were performed using the “clusterProfiler” R package (version 4.2.2) [15]. Further functional annotation and disease association analyses of the intersected LMRGs were conducted using the Metascape database (https://metascape.org/, accessed on 30 January 2026). Protein–protein interaction (PPI) and co-expression networks were constructed utilizing the STRING (https://string-db.org/, accessed on 30 January 2026) and GeneMANIA (https://genemania.org/, accessed on 30 January 2026) platforms, respectively.

### 2.3. Identification of Core Genes via Machine Learning

To identify the most diagnostically valuable feature genes, we employed three independent machine learning algorithms [16]. LASSO regression was implemented using the “glmnet” package (version 4.1.8). SVM-RFE analysis was performed using the “e1071” package (version 1.7.16), and the feature subset with maximum classification accuracy was retained. A random forest (RF) model was constructed using the “randomForest” package (version 4.7.1.1); genes with a mean decrease in Gini score >1 were selected as candidate features. Genes commonly identified by all three algorithms were defined as the core signature. To mitigate the risk of overfitting inherent in small cohorts, the diagnostic performance of the model was strictly evaluated using a stratified 5-fold cross-validation procedure. In each fold, a multivariate logistic regression model was fitted on the training set and applied to the held-out fold to generate out-of-fold predicted probabilities. Receiver operating characteristic (ROC) curves were constructed based on the aggregated out-of-fold predictions, and the area under the curve (AUC) with its 95% confidence interval was reported to quantify diagnostic performance in the SAP cohort.

### 2.4. Function and Immune Characteristics of Model Genes

To explore the potential molecular mechanisms of model genes in AP, we performed GSVA using the “GSVA” package (version 1.42.0) [17]. Immune cell infiltration was quantified with the CIBERSORT algorithm [18], and correlation analyses were subsequently conducted between core gene expression and immune cell abundance.

### 2.5. Immunofluorescence and Upstream Regulatory Network Analysis

To explore the subcellular localization of CES1, immunofluorescence staining images were retrieved from the Human Protein Atlas (HPA, https://www.proteinatlas.org/, accessed on 10 February 2026). To elucidate the potential transcriptional regulatory mechanisms of CES1, we utilized the TFTF platform (https://jingle.shinyapps.io/TF_Target_Finder/, accessed on 25 February 2026) to predict upstream transcription factors (TFs) [19].

### 2.6. scRNA-Seq and In Silico Knockout

The scRNA-seq data of mouse pancreatic tissues were obtained from the GSE279876 dataset (accessed on 15 March 2026) [20], comprising one control sample and one HAP sample. Quality control and normalization were performed using the “Seurat” package (version 4.3.0) [21]. Cells expressing fewer than 200 features or exhibiting more than 15% mitochondrial gene expression were excluded. Genes detected in fewer than 5 cells were also removed from subsequent analyses. Following quality control (QC), the data were log-normalized, and the top 1500 highly variable features were identified. Nonlinear dimensionality reduction was subsequently performed using t-SNE for visualization and downstream analysis. Cell clusters were annotated based on classical marker genes. Virtual knockout analysis of Ces1d was performed using the “scTenifoldKnk” package (version 1.0.3) [22]. The virtual knockout was conducted globally across all cell types, rather than being restricted to a single cell cluster.

### 2.7. Animal Model of HAP and Pharmacological Intervention

To evaluate the in vivo therapeutic potential of targeting CES1 in HAP, the CES1 inhibitor WWL113 was utilized in this study [23]. Male C57BL/6 mice (6 weeks old) were randomly assigned to three groups (*n* = 6): Con, HAP, and Treatment (WWL113). To establish the chronic hyperlipidemic background, mice in the HAP and WWL113 groups received intraperitoneal (i.p.) injections of Poloxamer 407 (P-407, 0.5 g/kg, MCE, Monmouth Junction, NJ, USA) every other day for 4 weeks. Pharmacologically, P-407 is a potent inhibitor of lipoprotein lipase (LPL) that prevents peripheral lipid clearance, thereby robustly mimicking chronic systemic hypertriglyceridemia [24,25]. On the day following the final P-407 injection, AP was induced. The mice were administered a total of 10 consecutive hourly i.p. injections of caerulein (100 μg/kg, MCE). Caerulein, a cholecystokinin analogue, hyperstimulates pancreatic acinar cells, causing premature intracellular zymogen activation and initiating localized pancreatic inflammation. Immediately following the final caerulein injection, a single i.p. injection of lipopolysaccharide (LPS, 10 mg/kg, MCE) was administered. LPS serves as a pathological “second hit” endotoxin to exacerbate the localized pancreatic injury into a severe and systemic inflammatory state. For pharmacological intervention, WWL113 (MCE) was dissolved in a vehicle containing DMSO, PEG 300, Tween-80, and sterile saline. WWL113 (30 mg/kg) was administered via a single i.p. injection 1 h prior to the first caerulein injection. Control mice received an equivalent volume of the vehicle alone via the same route. Mice were sacrificed 24 h after the initial caerulein injection to collect serum and pancreatic tissues. Model validation was confirmed by serum biochemical markers, histopathological examination, and local pro-inflammatory cytokine upregulation. All animal procedures received approval from the institutional animal ethics committee (No. XL2605050302).

### 2.8. Histological and Biochemical Analyses

Pancreatic tissues were fixed in 4% paraformaldehyde, embedded in paraffin, and sectioned for Hematoxylin and Eosin (H&E) staining. Histological damage, including edema, inflammatory cell infiltration, hemorrhage, and acinar necrosis, was scored blindly by two independent pathologists [26]. Serum levels of TG, total cholesterol (TC), amylase, lipase, and carboxylesterase (CarE) activity were measured using assay kits (Jiancheng, Nanjing, China) according to the manufacturer’s protocols.

### 2.9. Quantitative Real-Time PCR (qRT-PCR) and Western Blots

Total RNA was isolated using the TRIzol Universal total RNA extraction reagent (Accurate Biology, Changsha, China). RNA was reverse-transcribed by All-in-One First-Strand Synthesis MasterMix (Yugong Biolabs, Lianyungang, China). The relative mRNA expression fold changes between different groups were calculated using the 2^−ΔΔCt^ method. The primer sequences used in this study are listed in Supplementary Table S2.

Protein samples were separated by SDS-PAGE and then transferred onto PVDF membranes. The membranes were immunoblotted with primary antibodies, followed by secondary antibodies for 1 h. The primary antibodies used were as follows: CES1 antibody (Proteintech, Wuhan, China, 1:4000) and β-actin antibody (Abclonal, Wuhan, China, 1:5000).

### 2.10. Pseudo-Targeted Lipidomics Analysis

Lipid extraction from pancreatic tissues was performed using a methyl tert-butyl ether method. Chromatographic separation was achieved using an ACQUITY HSS C18 column (1.8 µm, 2.1 × 100 mm; Waters, Milford, MA, USA) held at 40 °C on a 4500 triple quadrupole mass spectrometer (SCIEX, Singapore), with 2 μL of sample injected. The mobile phases included (A) acetonitrile/water (60:40, *v*/*v*) and (B) isopropanol/acetonitrile (90:10, *v*/*v*), each supplemented with 5 mM ammonium formate. A gradient elution protocol was employed, beginning at 20% B and progressing to 95% B over 17.5 min, at a constant flow rate of 0.2 mL/min. The lipid metabolites were acquired in selective reaction monitoring (SRM) mode. Peak extraction and integration were performed using MultiQuant™ software (version B.06.00; SCIEX). Lipid identification was achieved by matching precursor/product ion pairs (SRM transitions) and retention times against reference databases, including LipidMaps, HMDB, the iPhenome™ SMOL library, and NIST 17 Tandem MS/MS. Detailed criteria regarding lipid metabolite identification are available in our earlier publication [26,27].

### 2.11. Data Processing and Analysis

Quantification was performed using the isotope-labelled internal standard method. The peak areas of annotated lipids were normalized to the corresponding internal standards to correct for potential matrix effects and instrumental response variations. For quality filtering, features with more than 20% missing values within any biological group were excluded, and remaining random missing values were imputed using the K-Nearest Neighbours algorithm. The normalized data matrix was subsequently log10-transformed and auto-scaled prior to statistical analysis using MetaboAnalyst 6.0 (https://www.metaboanalyst.ca/, accessed on 10 May 2026).

### 2.12. Statistical Analysis

All the analyses were performed using R (version 4.1.3) and GraphPad Prism (version 10.1.2). Data are presented as mean ± SD. Two-group and multi-group comparisons were evaluated using Student’s *t*-test and one-way ANOVA followed by Tukey’s post hoc test, respectively. Statistical significance was defined as *p*-value < 0.05.

## 3. Results

### 3.1. Identification of Genes Associated with the Comorbidity of SAP and Obesity

**Figure 1:**
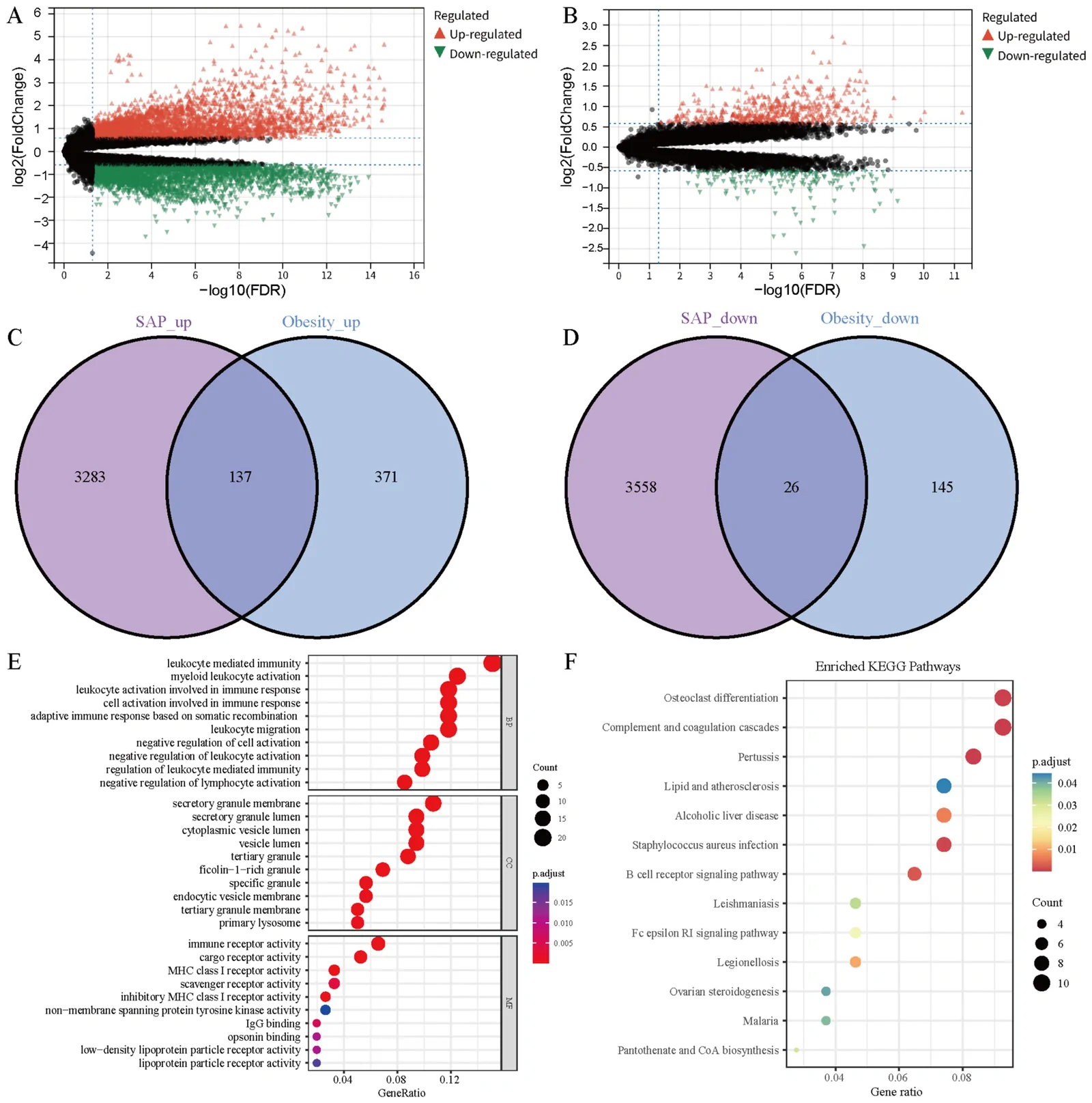
Identification and functional analysis of genes associated with the comorbidity of SAP and obesity: (**A**,**B**) Volcano plots of DEGs of the SAP samples (**A**) and the obesity samples (**B**). Red triangles indicate up-regulated genes, green inverted triangles indicate down-regulated genes, and black dots indicate non-significantly regulated genes. Dashed lines indicate the predefined cutoff values. (**C**,**D**) Venn diagrams of upregulated (**C**) and downregulated (**D**) genes across the two datasets. (**E**,**F**) GO (**E**) and KEGG (**F**) functional enrichment analyses of comorbidity DEGs.

To investigate the potential molecular mechanisms by which hypertriglyceridemia exacerbates AP, we conducted a joint analysis of the SAP samples and the obesity/hyperlipidemia samples. The volcano plot visually illustrates the overall distribution of DEGs between the two disease groups and the control group (Figure 1A,B). By cross-comparing the DEGs from the two groups, we identified a total of 163 comorbidity genes exhibiting consistent expression trends in both SAP and obesity samples, including 137 commonly upregulated genes (Figure 1C) and 26 commonly downregulated genes (Figure 1D). GO enrichment results (Figure 1E) show that these genes are primarily enriched in leukocyte-mediated immunity, secretory granule membrane, and immune receptor activity. KEGG pathway analysis (Figure 1F) further indicates that comorbidity genes are significantly enriched in pathways such as osteoclast differentiation, complement and coagulation cascade, and lipid metabolism and atherosclerosis.

### 3.2. Identification of Core Comorbidity Genes in Lipid Metabolism

**Figure 2:**
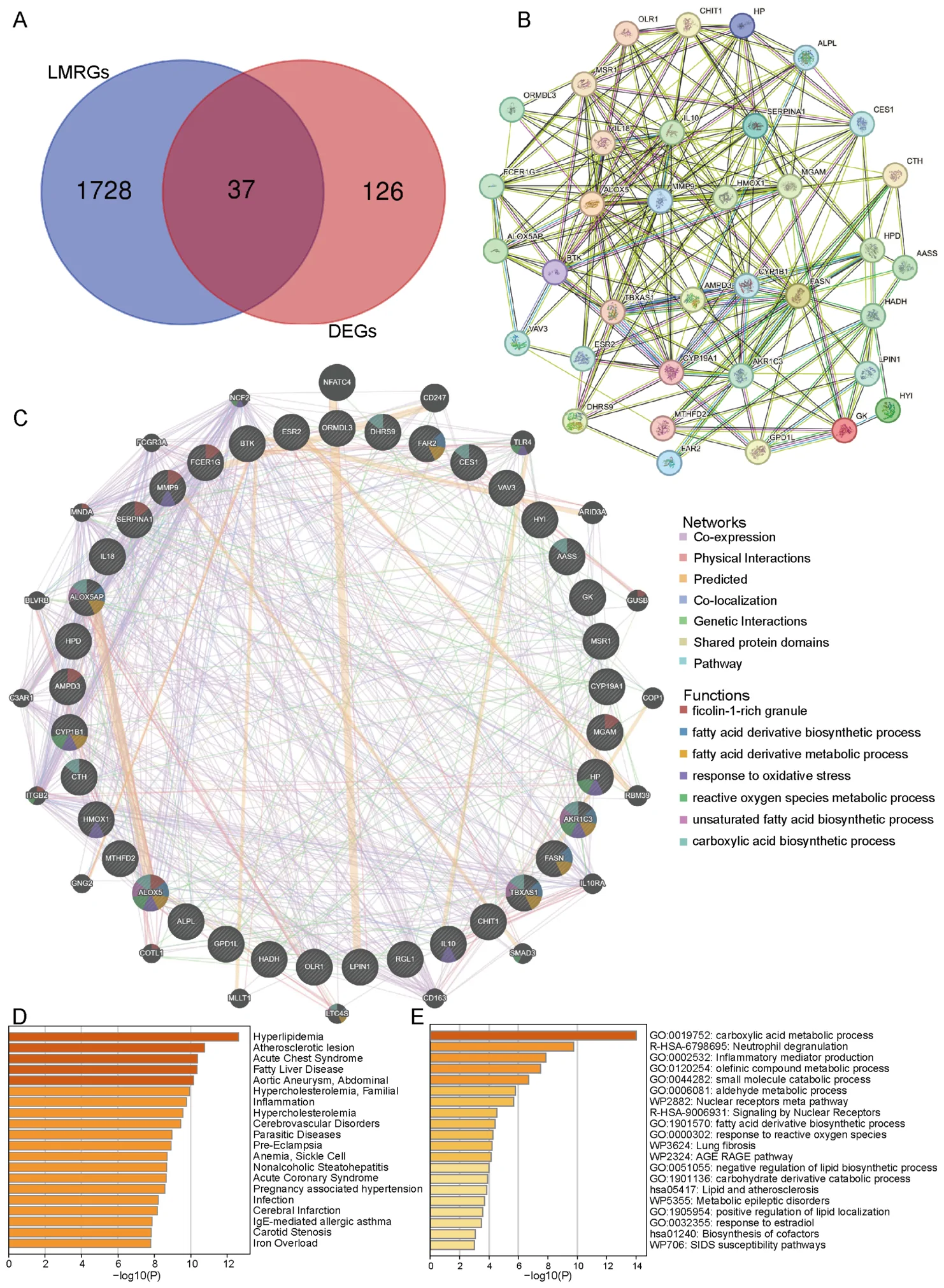
Screening of core lipid metabolism comorbidity genes and analysis of their interaction networks: (**A**) Venn diagram showing the intersection of comorbidity DEGs and LMRGs. (**B**,**C**) Protein interaction and co-expression networks based on the STRING (**B**) and GeneMANIA (**C**) databases. (**D**,**E**) Disease association (**D**) and functional enrichment (**E**) analyses based on the Metascape database.

Given the central role of lipotoxicity in the progression of HAP, we cross-screened the 163 comorbidity DEGs against a set of LMRGs. The results showed that 37 genes overlapped (Figure 2A). To elucidate the synergistic regulatory relationships among these genes, we constructed multidimensional molecular interaction networks. The PPI network and the GeneMANIA co-expression network jointly revealed a tightly structured regulatory module (Figure 2B,C). Disease enrichment analysis (Figure 2D) demonstrated that this gene set is highly enriched in metabolic and systemic inflammatory clinical phenotypes such as “hyperlipidemia,” “fatty liver disease,” and “inflammation.” At the functional and pathway enrichment level (Figure 2E), these core genes are extensively involved in key pathways such as “carboxylic acid metabolic processes,” “neutrophil degranulation,” and “production of molecular mediators involved in inflammatory responses.” These findings reveal a profound pathological coupling between specific lipid metabolism and neutrophil-mediated local immune storms.

### 3.3. Machine Learning Screening of Core Driver Genes

**Figure 3:**
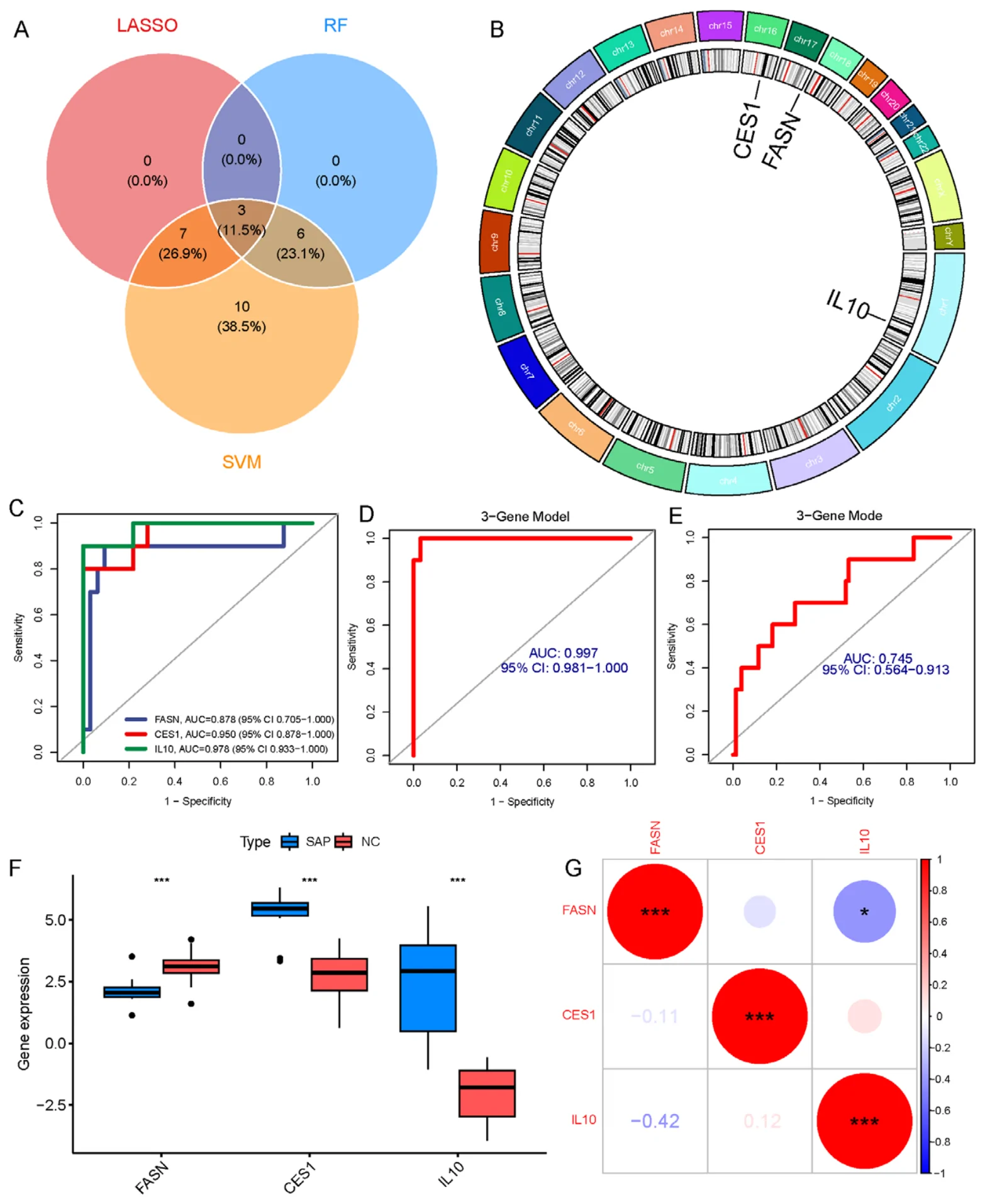
Development of diagnostic models and core gene expression features: (**A**) Venn diagram showing the intersection of feature subsets from the LASSO, SVM-RFE, and RF algorithms, identifying three core genes (FASN, CES1, IL10). (**B**) Chromosomal Circos map showing the locations of the three core genes. (**C**,**D**) ROC curves illustrating the diagnostic performance of the single-gene (**C**) and the combined model (**D**). (**E**) ROC curves illustrating the diagnostic performance of the signature in discriminating SAP from non-severe AP. (**F**) Box plots showing the relative expression levels of FASN, CES1, and IL10 in the control and SAP groups. (**G**) Correlation matrix among the core genes. * *p* < 0.05, *** *p* < 0.001.

To identify the core features with the highest diagnostic value from the candidate genes, we employed three independent machine learning algorithms for analysis. Ten feature genes were identified using the LASSO regression model (Supplementary Figure S1A,B). The SVM-RFE algorithm identified 26 feature genes with the highest cross-validation accuracy (Supplementary Figure S1C,D). The Random Forest algorithm identified 9 feature genes with importance scores greater than 1 (Supplementary Figure S1E,F). By intersecting the features identified by these three algorithms, we ultimately identified three overlapping core driver genes: FASN, CES1, and IL10 (Figure 3A). The chromosomal locations of these three core genes in the human genome are shown in Figure 3B. ROC curves showed that the AUCs for FASN, CES1, and IL10 were 0.878, 0.950, and 0.978, respectively (Figure 3C). Meanwhile, we constructed a three-gene combined model using multivariate logistic regression, which increased the AUC to 0.997 (95% CI: 0.981–1.000, Figure 3D). To further evaluate the clinical applicability and generalizability of this metabolic signature, we tested its performance in distinguishing SAP from non-severe AP (MAP and MSAP) within the same cohort. The model demonstrated moderate discriminative ability, achieving an AUC of 0.745 (95% CI: 0.564–0.913, Figure 3E). The box plots showed that both CES1 and IL10 were significantly upregulated under pathological conditions in SAP, while FASN was significantly downregulated (Figure 3F). Inter-gene correlation analysis revealed low collinearity among the three genes, suggesting the independence of the model features (Figure 3G).

### 3.4. Single-Gene GSVA Reveals Functional Heterogeneity of Core Genes

**Figure 4:**
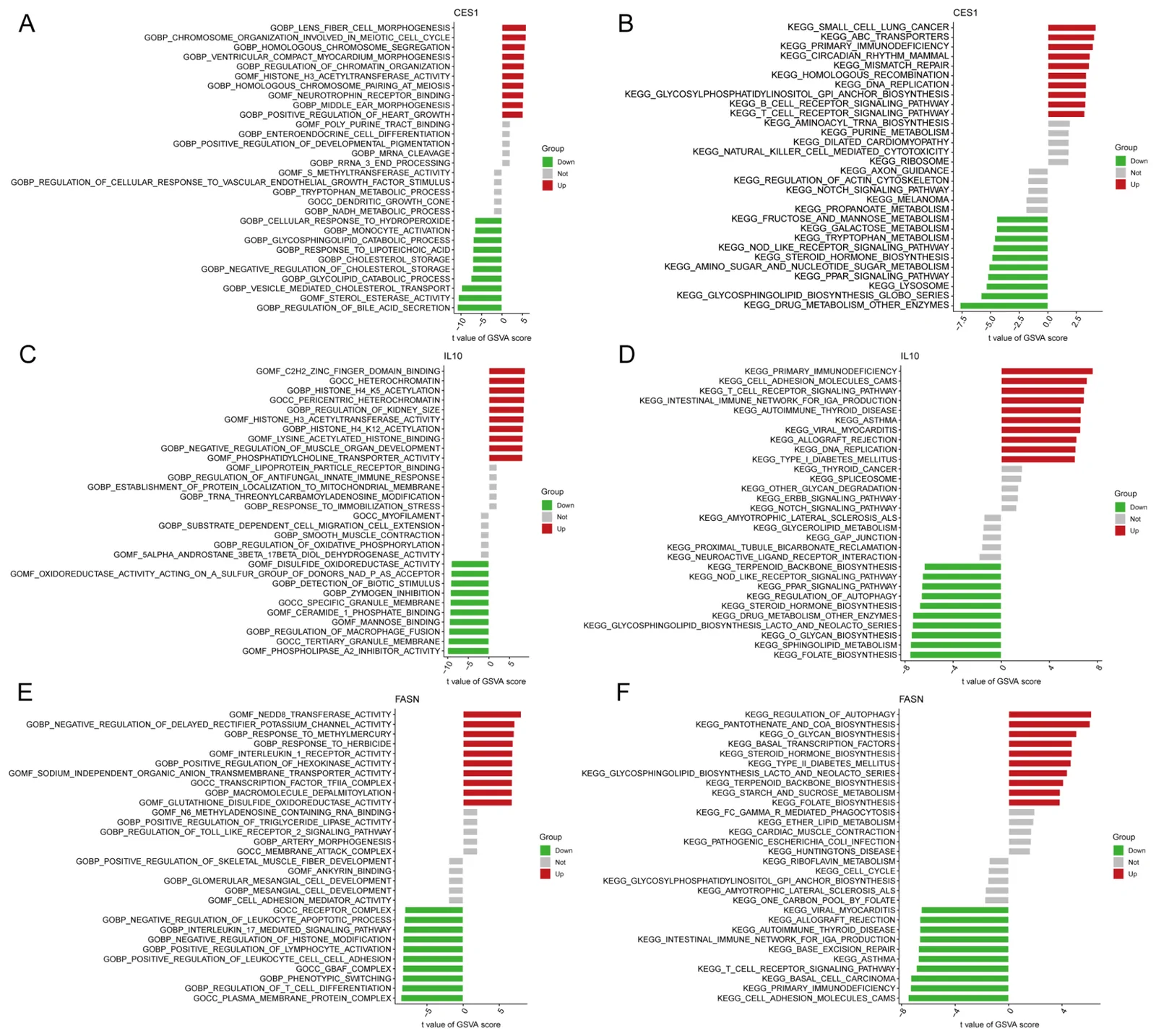
Single-gene GSVA of the core genes in AP. (**A**–**F**) Bar charts showing the functions and pathways in AP that are significantly associated with high/low expression phenotypes of CES1 (**A**,**B**), IL10 (**C**,**D**), and FASN (**E**,**F**).

To elucidate the specific biological functions of each core gene in SAP, we performed single-gene GSVA. For CES1, its enriched pathways included DNA replication, mismatch repair, and chromosome organization, alongside the activation of ABC transporters (Figure 4A,B). The high expression of the inflammatory marker IL10 was positively correlated with active immune-epigenetic remodeling. Its upregulated pathways encompassed both immune responses (e.g., T-cell receptor signaling and cell adhesion molecules) and chromatin modifications (Figure 4C,D). Conversely, the downregulation of FASN was strikingly linked to the suppression of critical immune and inflammatory cascades. The loss of FASN expression was accompanied by a marked inhibition of T-cell receptor signaling, leukocyte cell–cell adhesion, and IL-17-mediated signaling pathways (Figure 4E,F).

### 3.5. Characteristics of the Immune-Infiltrated Microenvironment of Core Genes

**Figure 5:**
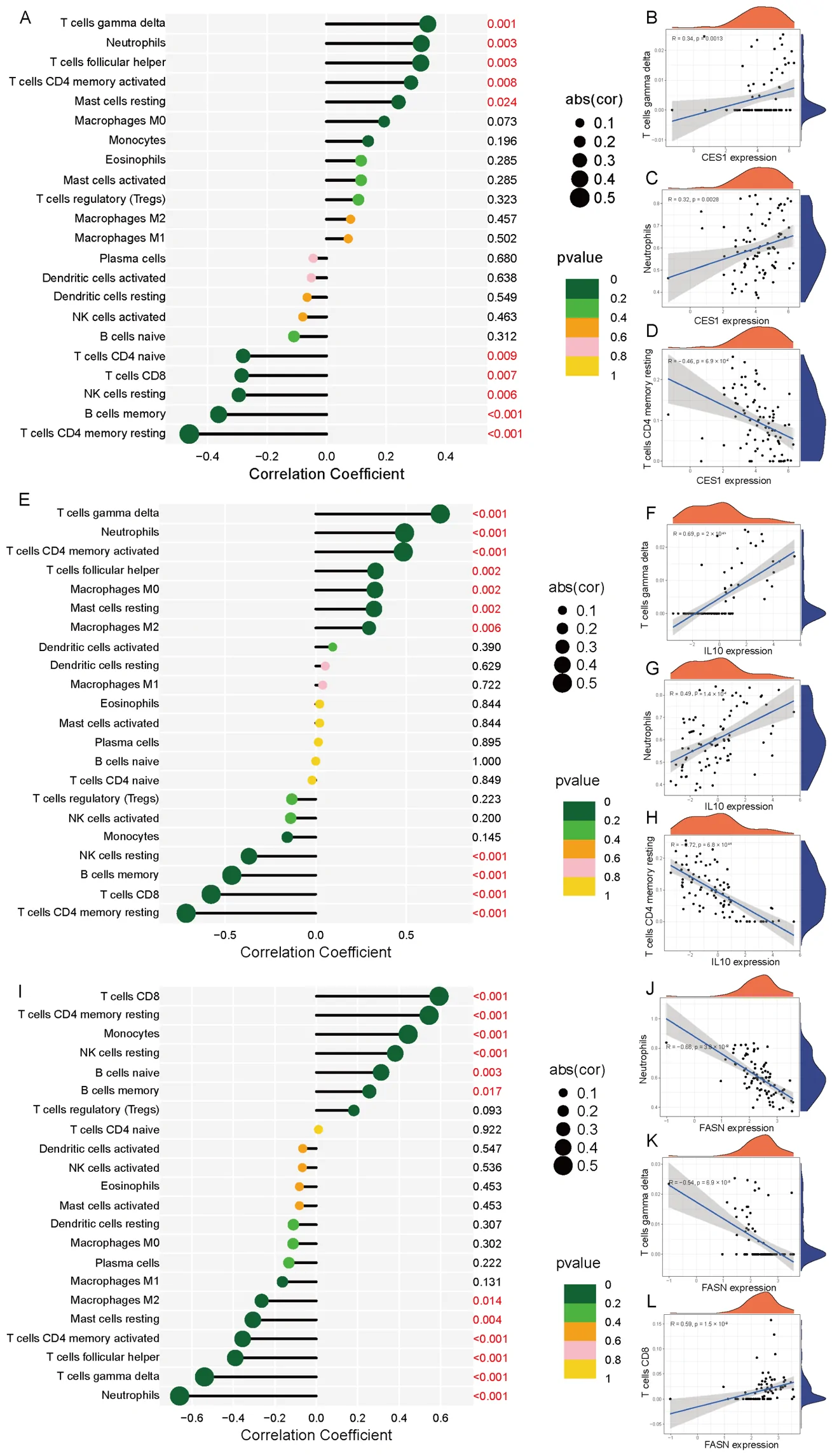
Immune infiltration landscape of the core genes in AP: (**A**) Correlations between different types of immune cells and CES1. (**B**–**D**) Analysis of the association between CES1 and γδ T cells (**B**), neutrophils (**C**), and resting CD4^+^ memory T cells (**D**). (**E**) Correlations between different types of immune cells and IL10. (**F**–**H**) Analysis of the association between IL10 and γδ T cells (**F**), neutrophils (**G**), and resting CD4^+^ memory T cells (**H**). (**I**) Correlations between different types of immune cells and FASN. (**J**–**L**) Analysis of the association between FASN and γδ T cells (**J**), neutrophils (**K**), and resting CD4^+^ memory T cells (**L**). Black dots represent individual samples. The blue line shows the linear regression line, and the gray shaded area indicates the 95% confidence interval of the regression. The orange marginal density plot at the top displays the distribution of gene expression values, and the blue marginal density plot on the right shows the distribution of immune cell proportion.

We then used the CIBERSORT algorithm to quantify the abundance of immune cell infiltration and performed correlation analyses with core genes. The results showed that the expression levels of CES1 and IL10 were significantly positively correlated with γδ T cells and neutrophils, while they were negatively correlated with resting CD4^+^ memory T cells (Figure 5A–H). Since FASN is downregulated in the disease state, its expression showed a very strong negative correlation with neutrophils and γδ T cells, while exhibiting a positive correlation with CD8 T cells (Figure 5I–L). Because both CES1 expression and neutrophil proportions were derived from the same bulk transcriptome, this correlation may be confounded by leukocyte composition and does not establish a causal role for CES1 in neutrophil recruitment. Nevertheless, this association is consistent with the neutrophil-dominant inflammatory phenotype characteristic of severe AP.

### 3.6. Single-Cell Analysis Reveals the Central Role of CES1

**Figure 6:**
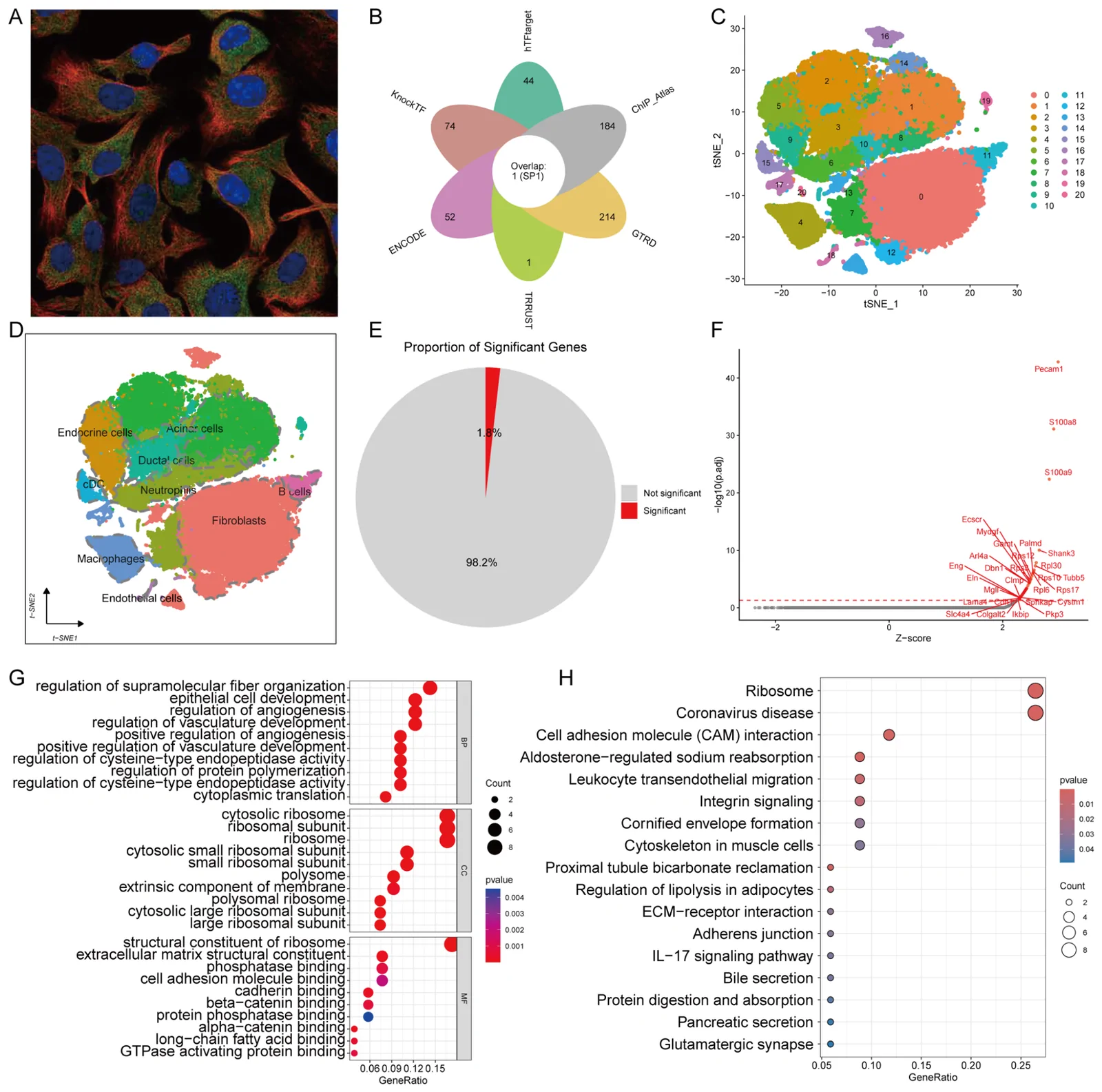
Upstream transcriptional regulatory network and in silico knockout of CES1: (**A**) Verification of the subcellular localization of CES1 using immunofluorescence data from the HPA. (**B**) Venn diagrams showing the predicted upstream TFs for CES1 across six databases. (**C**) t-SNE plot of scRNA-seq data delineating distinct cellular subclusters. (**D**) The t-SNE plot visualizing the 9 distinct cell subsets identified in the scRNA-seq data. (**E**) Pie chart summarizing the proportion of genes with significant expression changes following the virtual knockout of the Ces1d gene. (**F**) Volcano plot visualizing the downstream genes significantly affected by the Ces1d virtual knockout. Grey dots represent genes with non-significant enrichment, and the red dashed line denotes the significance threshold.(**G**,**H**) GO (**G**) and KEGG (**H**) enrichment analyses of the altered genes.

To identify the core regulator underlying lipotoxicity in HAP, we first referred to our previously published clinical lipidomics findings, which demonstrated that the serum metabolic profile of patients with HAP is characterized by a marked accumulation of FFAs and ceramides [28]. Consistent with the enhanced lipolytic phenotype, CES1, a major TG hydrolase, was significantly upregulated under pathological conditions. Given its pivotal role in TG hydrolysis and FFA production, CES1 was selected for subsequent investigation. Subcellular localization analysis using immunofluorescence data from the HPA revealed that CES1 was predominantly detected in the endoplasmic reticulum (Figure 6A). To identify its upstream regulator, we integrated predictions from six transcription factor databases. SP1 was identified as the only overlapping candidate transcription factor predicted to target CES1 across all six databases (Figure 6B).

To elucidate the functional network of CES1 at single-cell resolution, we analyzed scRNA-seq data from pancreatic tissue of HAP mice. t-SNE dimensionality reduction partitioned the pancreatic microenvironment into 20 functional subpopulations (Figure 6C) and accurately annotated nine major cell lineages (Figure 6D). Simulating the knockout of the Ces1d gene resulted in significant changes in 1.8% of global transcripts (Figure 6E). Differential analysis using a volcano plot revealed that virtual knockout of Ces1d significantly predicted downregulation of Pecam1, S100a8, and S100a9 (Figure 6F). Enrichment analysis revealed that these genes were enriched in leukocyte transendothelial migration, cell adhesion molecule interactions, and angiogenesis regulation (Figure 6G,H). These results suggest that in addition to lipid hydrolysis, CES1 expression patterns are closely associated with transcriptomic changes related to endothelial barrier damage and neutrophil chemotaxis. However, given the limitation of a single biological replicate in the scRNA-seq dataset, these in silico findings are strictly exploratory and associative.

### 3.7. Targeting CES1 Significantly Alleviates HAP-Induced Injury and Remodels Pancreatic Lipid Metabolism

**Figure 7:**
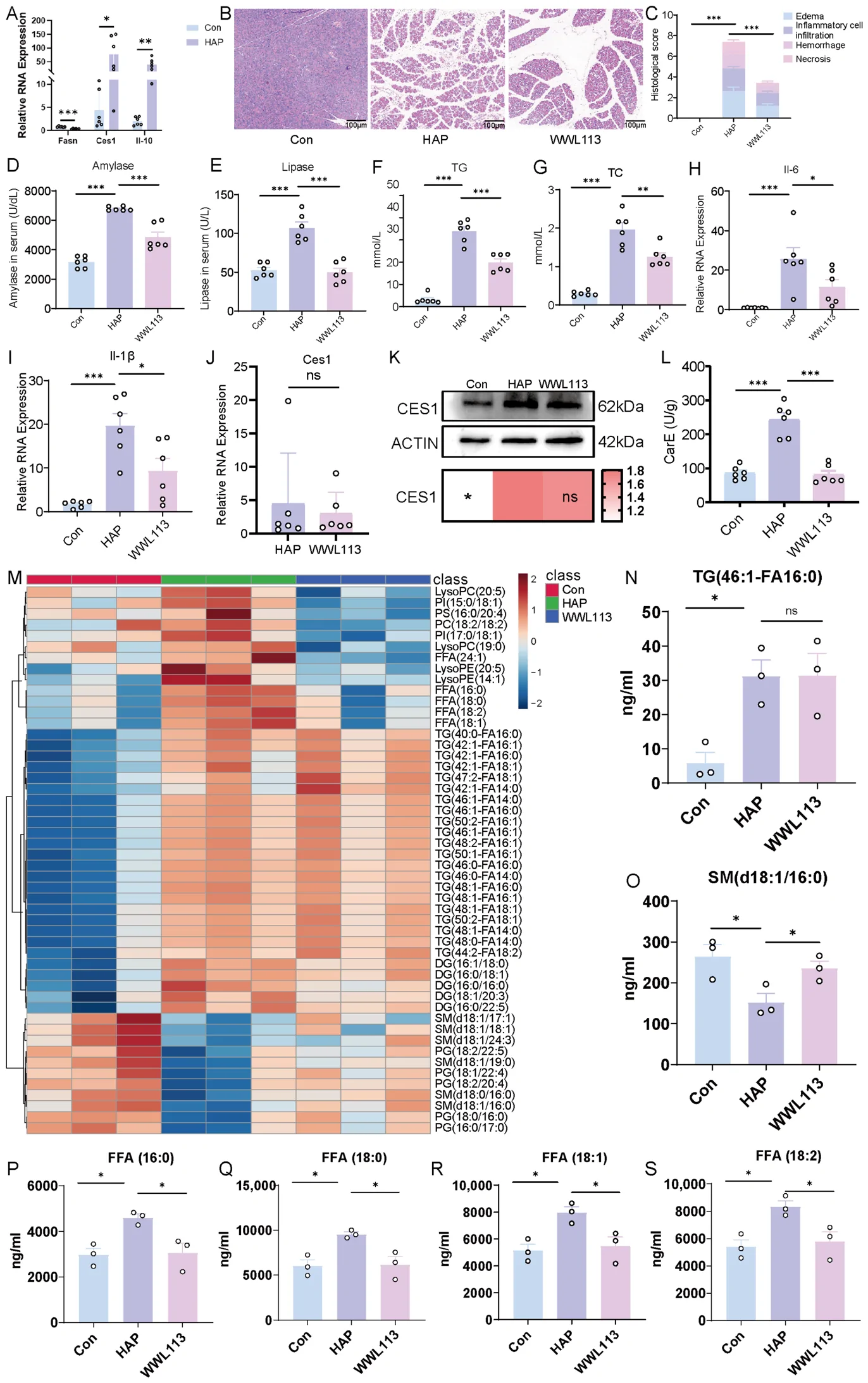
Efficacy and lipidomic reprogramming of the WWL113 in the HAP mouse model: (**A**) qRT-PCR analysis of the transcriptional expression of Fasn, Ces1d, and Il10 in the pancreas of the HAP group and the control group. (**B**,**C**) Representative H&E-stained images of pancreatic tissue and histopathological scores. (**D**–**G**) Measurement of serum amylase (**D**), lipase (**E**), TG (**F**), and TC (**G**) levels. (**H**,**I**) Relative expression levels of the inflammatory cytokines Il-6 and Il-1β in pancreatic tissue. (**J**) Relative mRNA expression of Ces1d between the HAP and WWL113 groups. (**K**) Western blot analysis and corresponding densitometric quantification of Ces1 protein expression in the pancreas. * *p* < 0.05, ns, not significant, vs. HAP. (**L**) Pancreatic CarE enzymatic activity assay, demonstrating profound functional inhibition by WWL113. (**M**) A clustering heatmap reveals the clearance of toxic FFAs, the sequestration of inert TGs, and the recovery of SM following WWL113 treatment. (**N**–**S**) Box plots comparing the relative abundances of the TG (46:1-FA16:0) (**N**), SM (d18:1/16:0) (**O**), FFA (16:0) (**P**), FFA (18:0) (**Q**), FFA (18:1) (**R**), and FFA (18:2) (**S**) across groups. ns, not significant, * *p* < 0.05, ** *p* < 0.01, *** *p* < 0.001.

To evaluate the prophylactic potential of CES1-targeted therapy in vivo, the HAP mouse model was established in this study. First, qRT-PCR results confirmed that, compared with the control group, Ces1d and Il10 were significantly upregulated in the pancreatic tissue of HAP mice, while Fasn was significantly downregulated (Figure 7A). This transcriptional profile was consistent with bioinformatics predictions, further validating the reliability of the results. Subsequently, we evaluated the in vivo efficacy of the CES1-inhibitor WWL113. Histological analysis revealed that the pancreas in the HAP group exhibited extensive acinar necrosis, severe edema, and inflammatory cell infiltration; however, intraperitoneal administration of WWL113 significantly alleviated these pathological lesions, with all histological scores showing a substantial decrease (Figure 7B,C). Consistent with these histological findings, WWL113 significantly decreased the abnormally elevated serum amylase and lipase levels, indicating an attenuation of pancreatic injury (Figure 7D,E). Meanwhile, WWL113 treatment significantly reduced serum TG and TC levels (Figure 7F,G), suggesting an improvement in lipid metabolic disturbances. In addition, WWL113 potently suppressed the transcriptional upregulation of the pro-inflammatory cytokines Il-6 and Il-1β in pancreatic tissue (Figure 7H,I).

To further assess target engagement in vivo, we assessed both the expression and functional activity of Ces1d in the pancreas. Interestingly, neither the mRNA transcription (Figure 7J) nor the protein abundance (Figure 7K) of Ces1d was reduced following WWL113 administration. However, the tissue CarE enzymatic activity assay revealed a profound functional suppression. The pathologically enhanced hydrolytic activity in HAP tissues was completely abrogated by WWL113 (Figure 7L). To decipher the pancreatic protective mechanism of WWL113, we performed lipidomics analysis. Heatmap and box plots indicated that WWL113 may reshape the HAP-associated lipid metabolome by inhibiting Ces1d activity, with TGs remaining at elevated levels while toxic FFAs were markedly reduced and structural lipid homeostasis was restored (Figure 7M–S).

## 4. Discussion

HAP is a severe subtype of AP characterized by rapid progression, a high rate of complications, and high mortality [29]. Clinically, obesity is a well-established risk factor that significantly exacerbates AP severity [30]. The systemic metabolic dysfunction inherent in obesity is intrinsically linked to hyperlipidemia, providing a substantial reservoir of circulating lipids that accelerates pancreatic injury [31]. Elevated serum TG levels are widely recognized to induce pancreatic lipotoxicity; however, TGs themselves are relatively inert storage molecules. Instead, FFAs released during TG hydrolysis are the principal mediators of lipotoxicity, disrupting organelle integrity, inducing intracellular calcium overload, and initiating inflammatory cascades [32,33,34]. However, the precise intracellular enzymatic mechanisms linking systemic hyperlipidemia to local pancreatic lipotoxicity remain largely unexplored.

In this study, by integrating transcriptomic cohorts of obesity/hyperlipidemia and SAP, we employed three machine learning algorithms to identify a robust comorbidity-associated signature comprising FASN, CES1, and IL10. Given the diverse clinical contexts of these discovery datasets, we currently position this signature as a broad metabolic-inflammatory vulnerability profile rather than a definitive HAP-specific mechanistic driver. Biologically, these three core genes represent key intersections between lipid metabolism and immune regulation, and all are closely associated with the pathogenesis of metabolic and inflammatory diseases. Their expression profiles, paired with single-gene GSVA analysis, collectively reveal profound metabolic and immune dysregulation in AP. FASN encodes the essential rate-limiting enzyme responsible for de novo fatty acid synthesis [35]. Previous studies have shown that FASN exhibits high activity in obesity and nonalcoholic fatty liver disease, leading to pathological lipid accumulation [36]. In our SAP cohort, FASN was significantly downregulated. This suppression may reflect a physiological negative feedback mechanism in which acinar cells attempt to halt endogenous lipid synthesis in an environment already flooded with exogenous lipids. In contrast, CES1, a potent intracellular TG and cholesterol ester hydrolase, was abnormally upregulated. CES1 has been studied for its role in hepatic lipid droplet mobilization and macrophage foam cell formation [37,38]. In the context of HAP, this FASN/CES1 transcriptional difference highlights a lethal maladaptive metabolic shift. Driven by a massive influx of exogenous TGs, the excessive activation of CES1 accelerates the breakdown of relatively inert TGs into highly toxic FFAs. Meanwhile, our GSVA results indicate that CES1 upregulation is associated with enhanced ABC transporter pathways, suggesting a strong compensatory effort by the cells to expel toxic lipids. Therefore, we hypothesize that this uncontrolled intracellular lipolysis constitutes the enzymatic engine of HAP-induced lipotoxic damage.

Beyond direct lipotoxicity, our analysis indicates that this abnormal lipid metabolism profoundly shapes the local inflammatory microenvironment. Immune infiltration analysis revealed that CES1 upregulation is closely associated with the accumulation of innate pro-inflammatory cells, particularly neutrophils and γδ T cells. To counteract this severe inflammatory storm and tissue damage, IL10 was found to be upregulated in SAP. IL10 is a classic multifunctional cytokine known for its immunosuppressive properties. In clinical SAP, elevated circulating IL10 is a well-documented phenomenon that often heralds the onset of the compensatory anti-inflammatory response syndrome and subsequent immune paralysis [39,40]. Our GSVA analysis further expands this observation, revealing that high IL10 expression is accompanied by the enrichment of immune receptor signaling and a broad spectrum of epigenetic remodeling pathways. This finding suggests that elevated IL10 levels may not merely represent a passive anti-inflammatory feedback mechanism, but may also be involved in complex systemic compensatory processes associated with epigenetic reprogramming to restore immune homeostasis during lipotoxic stress.

To construct a comprehensive regulatory axis for CES1, we integrated single-cell computational virtual knockout analysis with upstream transcription factor prediction. Our upstream network prediction suggests that SP1 may be a key transcription factor targeting CES1. SP1 is a classic stress response regulator deeply involved in lipid homeostasis and inflammatory signaling [41,42], indicating that the pathological upregulation of CES1 represents an active transcriptional response to acute lipotoxic crises. At the downstream effector level, simulated knockout of the Ces1d gene significantly downregulated endothelial junction markers (Pecam1) and key neutrophil chemotactic signaling proteins (S100a8 and S100a9); the affected genes were enriched in pathways controlling leukocyte migration through endothelial cells. Importantly, these virtual knockout results are exploratory and lack biological replication; therefore, they should be interpreted as hypothesis-generating predictions rather than evidence of a causal mechanism. Collectively, the predicted SP1–CES1–inflammation regulatory axis provides a potential framework linking CES1-associated lipid metabolic dysregulation with acinar injury, endothelial barrier dysfunction, and the amplification of neutrophil-driven local inflammation.

To validate these bioinformatics inferences, we utilized a P-407/caerulein-induced HAP mouse model and administered the CES1 inhibitor WWL113. Notably, pharmacological inhibition of CES1 significantly attenuated pancreatic necrosis, reduced serum amylase and lipase, and suppressed the expression of local pro-inflammatory cytokines. Our lipidomics analysis provided insight into the underlying protective mechanisms. Following WWL113 treatment, the abundance of intracellular TG and diacylglycerol (DG) remained elevated, while levels of toxic FFAs and membrane-damaging lysophospholipids dropped sharply. This distinct lipid remodeling pattern supports a model in which CES1-catalyzed TG hydrolysis promotes acute lipotoxicity through FFA generation, ultimately leading to acinar cell necrosis in HAP.

Despite these multi-omics validations, our study has certain limitations. First, our bioinformatics screening was primarily exploratory and hypothesis-generating. The relatively small sample size and limited availability of independent datasets may have resulted in an overestimation of the diagnostic performance, particularly the AUC, and the lack of validation in an independent, large-scale, prospective clinical cohort remains an important limitation. Second, the single-cell virtual knockout analysis was based on a public dataset containing only one biological replicate per condition, rendering these computational inferences strictly associative. Third, future investigations incorporating normolipidemic AP models are required to precisely delineate the hyperlipidemia-specific lipotoxic contributions from the general inflammatory responses. In addition, because WWL113 was administered systemically via intraperitoneal injection, the relative contributions of systemic metabolic effects and local pancreatic CES1 inhibition could not be definitively distinguished. Further pharmacokinetic, tissue-specific target-engagement, and functional studies will be required to clarify the local versus systemic mechanisms of WWL113. Finally, although WWL113 provided pharmacological evidence supporting Ces1d involvement, its potential off-target effects within the murine carboxylesterase family warrant future validation using cell-type-specific genetic knockout or rescue approaches.

## 5. Conclusions

This study identified a three-gene biomarker signature for SAP and highlighted CES1 as a key intracellular effector of lipotoxicity. By actively hydrolyzing excess TGs into toxic FFAs, CES1 links systemic metabolic dysfunction to localized acinar necrosis and neutrophil-driven inflammation. Targeting this lipolytic pathway offers pharmacological support for CES1-targeted prophylaxis in HAP.

## Data Availability

The datasets analyzed in this work can be found in the GEO database (https://www.ncbi.nlm.nih.gov/gds, (accessed on 28 August 2026)). The lipidomic data are not publicly available due to privacy. Further inquiries can be directed to the corresponding author.

## Supplementary Materials

The following supporting information can be downloaded at https://www.mdpi.com/article/10.3390/metabo16090644/s1, Figure S1: Machine learning algorithms identify core genes; Table S1: A gene list of LMRGs; Table S2: Primers used in the PCR analysis.
