# Supplementary material for: Integrated Transcriptomics and Lipidomics Identify CES1-Mediated Maladaptive Lipolysis as a Key Target of Hyperlipidemic Acute Pancreatitis
Source: Metabolites. 2026 Sep 3;16(9):644. doi: 10.3390/metabo16090644 (PMC13609043; doi:10.3390/metabo16090644)
Supplement: Supplementary file 1 [file metabolites-16-00644-s001.zip › metabolites-4467211-supplementary.pdf]

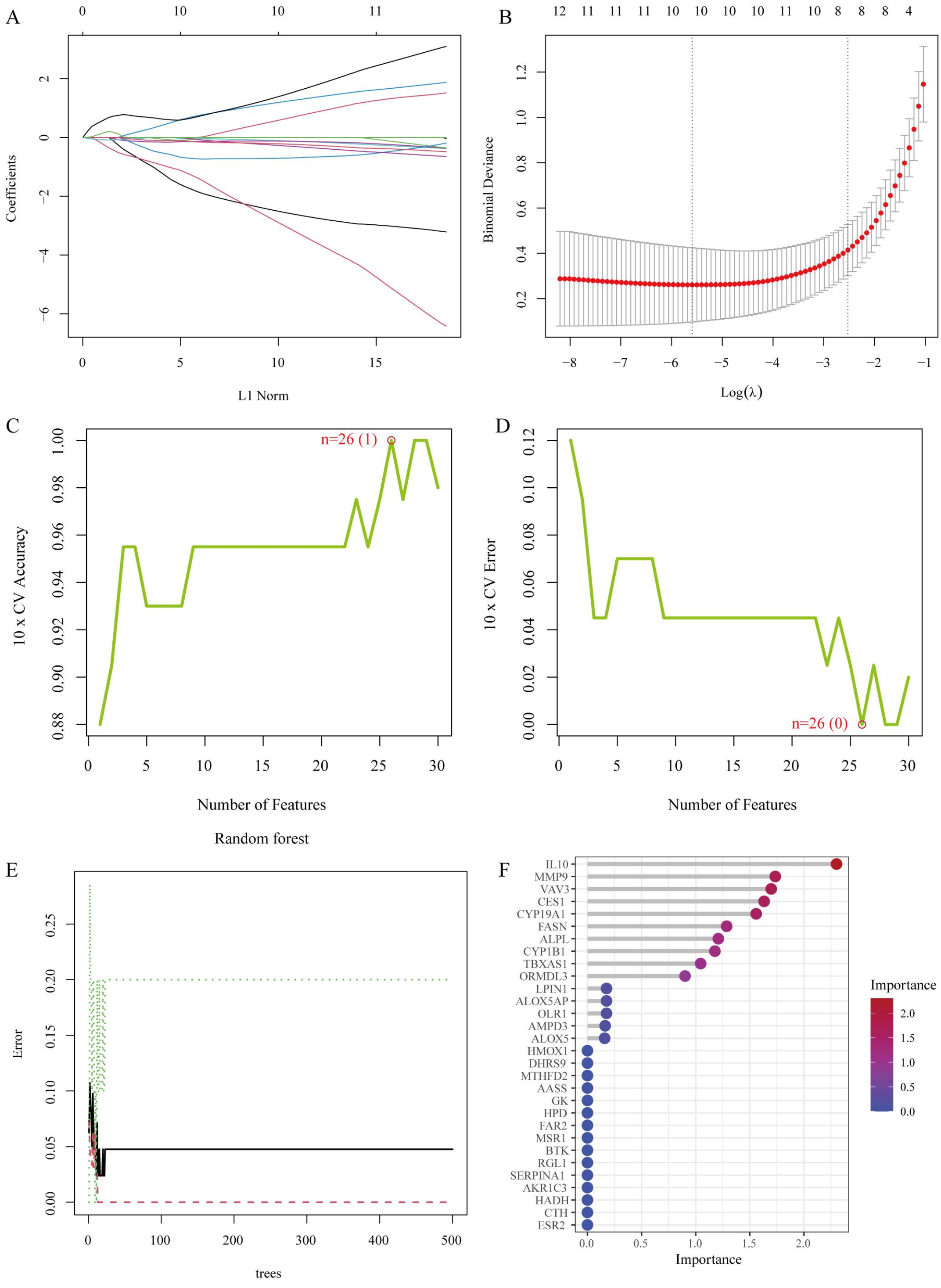


Supplementary Figure S1. Machine learning algorithms identify core genes. (A–B) Feature selection using LASSO regression and selection of optimal parameters via cross-validation. (C–D) Classification accuracy and cross-validation error plots for the SVM-RFE algorithm. (E–F) Error rate distribution for the RF algorithm and feature importance ranking based on the Gini coefficient.

Table S1. A gene list of LMRGs.

| |
|---|
| CYP2E1 |
| ESRRA |
| IDI2 |
| CYP11B1 |
| ACACB |
| ARSG |
| GRHL1 |
| SLC44A5 |
| PITPNM2 |
| PIP4K2B |
| MOGAT1 |
| STARD6 |
| CDK8 |
| THEM4 |
| DGKH |
| PLB1 |
| MFSD2A |
| DGKB |
| THRSP |
| PPARGC1B |
| SP1 |
| CBR4 |
| PIK3C2A |
| PIKFYVE |
| ACER1 |
| CEL |
| CYP39A1 |
| ARSB |
| SPTLC1 |
| HSD17B12 |
| ZNF638 |
| CERS3 |
| CYP19A1 |
| NR1H4 |
| HILPDA |
| SAR1B |
| MBTPS2 |
| DGKE |
| OSBPL1A |
| PLA2G4C |
| CYP2U1 |
| HMGCLL1 |
| PRKD3 |

ESYT3
ACOT11
ACAT1
PTGS2
SYNJ2
LPGAT1
SLC44A4
B4GALNT1
ALOX15B
ASAH1
CYP4B1
GDPD1
AGK
TRIB3
ESYT2
AKR1C1
HEXA
ACSF3
PLD1
SQLE
PCCB
FAR2
FAR1
PRKAB2
UBE2I
KDSR
PPARA
PIK3R6
SREBF1
NFYC
CHPT1
AWAT1
PLEKHA8
TXNRD1
CEPT1
CCND3
CYP17A1
GPD1
OSBP
HSD17B4
INPP4B
ALB
ALDH3B1
FABP6

PLD4
ELOVL5
PIK3R2
NCOA6
ACBD5
INPP5D
CCNC
ARSK
PPARGC1A
PIK3C3
ABCA1
TBL1X
BDH1
ABCB4
ARF3
STARD7
PTGES3
CTSA
ACADVL
PPP1CC
RAN
SCD
PPP1CA
NCOR1
DHCR24
PSAP
RAB14
PPT1
HADHB
HADH
PLD3
GPX4
ACLY
ECHS1
LGMN
SREBF2
CDIPT
AKR1B1
FDPS
SCP2
CSNK2B
PPP1CB
ALDH2
PTDSS1

MAPKAPK2
INPPL1
ALDH9A1
MBTPS1
INSIG1
ACSL3
RELA
DHCR7
LBR
LPCAT1
PON2
AKR1A1
HEXB
GNPAT
ACSL1
MED13
ACAA2
ACAA1
ALDH3A2
NDUFAB1
TSPO
CPNE3
PLIN3
CREBBP
MTMR3
FADS2
EP300
LSS
CDK4
LYPLA2
GGPS1
SEC24C
ACADS
SEC24D
RXRA
DBI
CYP1B1
DECR1
LPIN2
ACADM
PITPNB
HMGCR
VAPB
CSNK1G2

GDE1
MED14
EBP
PRKACB
PIK3R3
HMGCL
OXCT1
INPP5K
LPCAT3
SACM1L
SEC24B
PRKACA
AHR
GPX2
AGT
FHL2
ARNT2
STARD3
LYPLA1
MVD
CAV1
TGFB1
SPTLC2
MTMR2
PHYH
DGKA
OCRL
MED1
GCDH
MED12
PMVK
LPL
ADIRF
CSNK2A2
INPP5F
CPT1A
FDX1
PLA2G2A
FIG4
SLC25A20
DGAT1
PNPLA6
STS
PITPNM1

PCCA
PIK3CD
HPGD
NR1H3
MORC2
CEBPD
CYP27A1
FABP4
CEBPA
PTEN
MVK
ME1
SMARCD3
MTM1
NFYA
HSD11B2
GSTM4
ALOX5AP
PTPN13
PCYT1A
ACOT8
GLIPR1
FAAH
CHKA
ACOX3
VDR
ELOVL6
CPT2
CYP11A1
SEC23A
MED7
PIK3CA
GALC
LCAT
ARSA
ALOX5
APOA1
PLA2G15
PIK3C2B
HSD3B1
STAR
INPP4A
ACOT13
CROT

HMGCS2
IDI1
CGA
NRF1
SRD5A1
PLA2G6
INPP5E
HSD17B2
MTMR9
UGCG
ALDH3B2
GC
CLOCK
FABP7
CYP2J2
SLC22A5
PLEKHA6
PTGS1
ABCD1
PEX11A
EHHADH
EGR2
MTF1
ACADSB
ACOX2
ACHE
AGPS
HSD11B1
BCHE
NPAS2
PIP4K2A
GGT5
SMPD2
PIP5K1B
ALAS1
CYP27B1
CDS1
LRP2
POMC
NCOA2
FABP3
CYP1A1
SLC27A2
HMGCS1

HSD17B1
CRAT
PRKD1
FABP1
PNLIP
PLIN1
ACSM3
DPEP1
CYP3A4
ANKRD1
ACADL
CSNK2A1
RAB5A
PI4KB
CYP4F11
PLA2G5
WNT10B
ADH1C
SULT2A1
HSD3B2
PLA2G1B
SERPINA6
LIPF
PON1
SLCO1B3
PIK3CG
DGKG
BMX
ACSBG1
LTC4S
CD36
CYP24A1
GLB1L
MED22
SLC2A4
LIPC
PNLIPRP1
HPGDS
PCYT1B
CUBN
DGKI
ARSD
BAAT
CPNE1

CH25H
SRD5A2
NEU3
MED20
HSD17B3
MED6
LEP
SLC10A2
AKR1D1
TNF
ALPI
ADIPOQ
SLC10A1
ALOX12
PRKACG
SLCO1A2
ALOX15
ALOX12B
CYP7B1
GK
PTGES
PIP5K1A
CYP7A1
PLA2R1
DEGS1
FABP2
MOGAT2
ADH6
DGKZ
DGKQ
SYNJ1
CYP1A2
PEMT
ACOX1
NCOA3
ALOXE3
PRKAA2
NCOR2
CYP2R1
FDXR
ADH1A
ACOT7
PPARD
DGKD

MED25
TBXAS1
PTGIS
CYP2C8
ABCC3
OSBPL7
LIPE
ABCB11
TNFAIP8
UGT8
SGPL1
PCK1
PPARG
WNT1
HADHA
FDFT1
VAPA
ADH5
ESYT1
NEU1
ALDH7A1
KPNB1
NCOA1
NR2F2
PLIN2
MSMO1
RXRB
AKR1C3
MBOAT7
KLF5
CBR1
OSBPL2
NFKB1
NSDHL
PRKD2
PI4K2A
GPS2
SIN3B
EPHX2
SMPD1
INSIG2
RGL1
PCYT2
ACAT2

ADH1B
OSBPL3
PLD2
ALDH1B1
GM2A
PNPLA4
ECI1
ARNTL
SPHK2
GPD2
SLC25A1
TM7SF2
PLA2G4A
SLCO1B1
CPNE6
RORA
CYP4F2
ADH7
AKR1C4
CYP4F8
AGPAT2
ARNT
MGLL
ACSL6
B3GALNT1
SC5D
PTGDS
CDK19
SLC25A17
PNLIPRP2
ACACA
MED13L
FASN
PIK3R1
LPIN1
MTMR4
SCAP
SBF1
CERS6
SPTSSA
CEBPB
GPD1L
PIP5K1C
OSBPL8

CHD9
PIK3CB
DDHD2
PNPLA2
PIK3R4
CDS2
PIAS4
SEC24A
SGMS1
ABHD3
LPCAT4
MED8
MCAT
MBOAT2
GDPD5
MTMR1
G0S2
HSD17B8
ZNF467
INPP5J
PON3
ELOVL2
ABHD5
STARD5
CYP2C9
MTMR6
GLA
ARSF
TNFRSF21
CYP11B2
PIK3C2G
PRKAG2
MED24
MED27
GK2
AGPAT1
MTMR7
NUDT7
CYP2C19
HDAC3
VAC14
OSBPL10
PLEKHA2
MED4

THRAP3
HSD17B11
ECI2
OSBPL9
PTGES2
AGPAT5
NFYB
NR1H2
RUFY1
MID1IP1
BDH2
ACSL5
MED9
CERK
AACS
MED28
PLBD1
ORMDL2
ABHD4
MECR
PCTP
ACP6
SRD5A3
ACSF2
MED23
MLYCD
CERS4
PIP4K2C
PLEKHA4
ETNK1
PLEKHA1
HSD17B14
LIPG
TGS1
SPHK1
GLTP
ETNK2
MED31
PGS1
CIDEC
ACBD4
FA2H
DPEP2
APOA2

ELOVL4
PLA1A
ACSS3
DECR2
AGPAT4
SMPD3
CPNE7
GDPD3
AGPAT3
SLC27A5
TIAM2
ARSJ
OLAH
ACAD10
DPEP3
TPTE
SCD5
KLF4
MTMR10
CYP46A1
PLA2G2D
SPTLC3
PIK3R5
ADPRM
PNPLA3
DHRS7B
CPTP
PLA2G3
HAO2
PLEKHA5
MTMR12
PTDSS2
ETNPPL
ANGPTL4
PLA2G12A
PECR
CHAT
PITPNM3
SGPP1
CIDEA
NEU2
PLA2G2E
PLA2G2F
MED16

TBL1XR1
MED17
ACOT9
ACSBG2
LDLRAP1
MTMR14
MED15
CERS2
SLC27A3
SLC44A1
ACER3
HSD3B7
LPCAT2
NEU4
STARD3NL
STARD10
ORMDL1
HACL1
ARV1
MED10
ORMDL3
PNPLA8
PLEKHA3
OSBPL5
TNFAIP8L2
ADH4
OSBPL6
GBA2
CRLS1
APOA5
DGAT2
PLA2G12B
SLC44A2
GPCPD1
CERS5
SUMF2
SIN3A
ACBD6
GPAM
SPNS2
MED29
PTPMT1
SAMD8
DDHD1

SBF2
MCEE
MED19
STARD4
SLC27A1
FAM120B
FITM2
SUMF1
MED11
LCLAT1
SGMS2
ELOVL7
MBOAT1
TNFAIP8L1
CPT1C
MED26
EBF1
MED30
PPM1L
SLC44A3
HELZ2
PNPLA7
PTGR1
SLC51A
AHRR
ACOT4
FITM1
TMEM86B
ENPP6
ARSI
PTGR2
FAAH2
SLC51B
ARF1
CYP8B1
PNPLA5
ELOVL3
MOGAT3
NUDT19
LIPH
PLA2G4F
PHOSPHO1
MMAA
ACOT12

ENPP7
GLYCTK
SGPP2
SPTSSB
TECRL
LIPI
PLD6
CYP4F22
ATP1A1
ATP1A2
ATP1A3
ATP1A4
ATP1B1
ATP1B2
ATP1B3
ATP1B4
FXYD2
FXYD4
IGF1
INS
INSR
IRS1
IRS2
IRS4
KCNJ1
KRAS
MAPK1
MAPK3
NEDD4L
NR3C2
PDPK1
PRKCA
PRKCB
PRKCG
SCNN1A
SCNN1B
SCNN1G
SFN
SGK1
SLC9A3R2
ALDH6A1
IMPA1
IMPA2
INPP1

INPP5A
INPP5B
IPMK
IPPK
ISYNA1
ITPK1
ITPKA
ITPKB
MINPP1
MIOX
PI4KA
PLCB1
PLCB2
PLCB3
PLCB4
PLCD1
PLCD3
PLCD4
PLCE1
PLCG1
PLCG2
PLCZ1
TPI1
AKR1B10
CYP2C18
CYP3A43
CYP3A5
CYP3A7
JMJD7-PLA2G4B
PLA2G10
PLA2G2C
PLA2G4B
PLA2G4E
ABAT
ACSS1
ACSS2
HIBCH
LDHA
LDHAL6A
LDHAL6B
LDHB
LDHC
MMUT
SUCLA2

SUCLG1
SUCLG2
SUCLG2P2
ACSL4
APOC3
AQP7
CPT1B
CYP4A11
CYP4A22
FABP5
ILK
MMP1
OLR1
PCK2
PLTP
RXRG
SLC27A4
SLC27A6
SORBS1
UBC
UCP1
UGDH
UGT1A1
UGT1A10
UGT1A3
UGT1A4
UGT1A5
UGT1A6
UGT1A7
UGT1A8
UGT1A9
UGT2A1
UGT2A3
UGT2B10
UGT2B11
UGT2B15
UGT2B17
UGT2B28
UGT2B4
UGT2B7
CBR3
CYP2B6
CYP4F3
GGT1

GGT6
GGT7
GPX1
GPX3
GPX5
GPX6
GPX7
LTA4H
AKT1
AKT2
AKT3
BTK
CSF2
FCER1A
FCER1G
FYN
GAB2
GRB2
HRAS
IL13
IL3
IL4
IL5
LAT
LCP2
LYN
MAP2K1
MAP2K2
MAP2K3
MAP2K4
MAP2K6
MAP2K7
MAPK10
MAPK11
MAPK12
MAPK13
MAPK14
MAPK8
MAPK9
MS4A2
NRAS
PRKCD
PRKCE
RAC1

RAC2
RAC3
RAF1
SOS1
SOS2
SYK
VAV1
VAV2
VAV3
AMDHD2
CHIA
CHIT1
CMAS
CYB5R1
CYB5R3
FCSK
FPGT
GALE
GALK1
GALK2
GALT
GCK
GFPT1
GFPT2
GFUS
GMDS
GMPPA
GMPPB
GNE
GNPDA1
GNPDA2
GNPNAT1
GPI
HK1
HK2
HK3
MPI
NAGK
NANP
NANS
NPL
PGM1
PGM2
PGM3

PMM1
PMM2
RENBP
UAP1
UGP2
UXS1
CHKB
GPAT2
GPAT3
GPAT4
PISD
PLPP1
PLPP2
PLPP3
TAZ
ACYP1
ACYP2
DLAT
DLD
GLO1
GRHPR
HAGH
HAGHL
LDHD
MDH1
MDH2
ME2
ME3
PC
PDHA1
PDHA2
PDHB
PKLR
PKM
DPM2
GPAA1
GPLD1
PGAP1
PIGA
PIGB
PIGC
PIGF
PIGG
PIGH

PIGK
PIGL
PIGM
PIGN
PIGO
PIGP
PIGQ
PIGS
PIGT
PIGU
PIGV
PIGW
PIGX
PIGY
PIGZ
ACO1
ACO2
AFMID
CS
HAO1
HYI
MTHFD1
MTHFD1L
MTHFD2
MTHFD2L
PGP
AOX1
BST1
CD38
ENPP1
ENPP3
NADK
NADSYN1
NAMPT
NMNAT1
NMNAT2
NMNAT3
NMRK1
NNMT
NNT
NT5C
NT5C1A
NT5C1B
NT5C2

NT5C3A
NT5E
NT5M
NUDT12
PNP
QPRT
AWAT2
TKFC
ENPP2
PAFAH1B1
PAFAH1B2
PAFAH1B3
PAFAH2
PLA2G7
ACSM1
ACSM2A
ACSM4
ACSM5
ALDH5A1
GAD1
GAD2
L2HGDH
OXCT2
ACP1
ACP2
ACP3
ACP4
ACP5
FLAD1
PHPT1
RFK
TYR
BPNT1
CHST11
CHST12
CHST13
PAPSS1
PAPSS2
SULT1A1
SULT1A2
SULT1A3
SULT1A4
SULT1E1
SULT2B1

SUOX
ADO
CDO1
CSAD
ACER2
ASAH2
B4GALT6
DEGS2
GAL3ST1
GBA
GLB1
SMPD4
ALDH1A1
ALDH1A2
BCO1
CYP26A1
CYP26B1
CYP26C1
CYP2A13
CYP2A6
CYP2A7
DHRS3
DHRS4
DHRS4L2
DHRS9
LRAT
RDH10
RDH11
RDH12
RDH16
RDH5
RDH8
RETSAT
RPE65
ABCD2
ABCD3
ABCD4
AGXT
AMACR
CAT
DAO
DDO
ECH1
GSTK1

IDH1
IDH2
MPV17
MPV17L
NOS2
PAOX
PEX1
PEX10
PEX11B
PEX11G
PEX12
PEX13
PEX14
PEX16
PEX19
PEX2
PEX26
PEX3
PEX5
PEX6
PEX7
PIPOX
PRDX1
PRDX5
PXMP2
PXMP4
SOD1
SOD2
XDH
AOC2
AOC3
CNDP1
DPYD
DPYS
SMS
SRM
UPB1
ALDH1A3
ALDH3A1
CYP2D6
FMO1
FMO2
FMO3
FMO4

FMO5
GSTA1
GSTA2
GSTA3
GSTA4
GSTA5
GSTM1
GSTM2
GSTM3
GSTM5
GSTO1
GSTO2
GSTP1
GSTT1
GSTT2
GSTZ1
MAOA
MAOB
MGST1
MGST2
MGST3
APOE
CETP
LDLR
APOB
ABCG5
ABCA3
ABCG8
MTR
MMACHC
FADS1
SLC19A3
MTTP
SFTPB
LINC01672
SFTPC
PCSK9
MTRR
IL6
TPK1
MMADHC
CRP
NPC1
MTHFR

LIPA
ACE
CSF2RA
ANGPTL3
OPA3
CBS
BSCL2
APOC2
MMAB
GHRL
GPT
SCARB1
LEPR
ETFDH
HNF4A
SLC25A19
GBA1
OTC
H19
SOD2-OT1
RETN
SIRT1
LPA
CES1
LMNA
BDNF-AS
GCG
SERPINE1
MIR122
MIR33A
SMAD5-AS1
TPMT
G6PD
TLR4
APOA4
NPC2
TAFAZZIN
SLC17A5
G6PC1
MLXIPL
TP53
ABCG1
BGLAP
MT-TP

IL1B
ESR1
LDAH
SHBG
CHKB-CPT1B
AUP1
COG2
CP
MIR7-3HG
ADRB3
CCL2
NOS3
APP
CNR1
SECISBP2
UCP2
LMBRD1
NPC1L1
SLC2A1
TMX2-CTNND1
ABCB1
ETFA
PRKAA1
PAH
IL10
FGF21
C3
COMMD1
MEG3
RBP4
STARD13
SLC25A13
MIR21
MTOR
NR1I2
DPP4
UCP3
TCF7L2
REN
HIF1A
HFE
SNCA
ERLIN2
CYP21A2

HCFC1
PRODH
SOAT2
ADRB2
SEC14L2
MT-ATP6
ETFB
GSR
F2
CFTR
SOAT1
ADSL
MIR33B
FGF23
MIR17
MIR126
TF
NAT2
ADA
MT-TL1
STARD8
HSD17B10
HNF1A
ERLIN1
SLC25A4
POR
BTD
PRKAG3
SERPINA3
ADIPOR2
MIR34A
TRU-TCA1-1
STARD9
APOC1
ASS1
MCOLN1
MT-CYB
LDAF1
PLIN5
CSF2RB
GCKR
NR1I3
EPHX1
CASR

CERNA3
FTO
COMT
PRKAB1
MIR155
QDPR
PTH
GALNT2
HMOX1
ACAD9
NR3C1
SCO2
EMSLR
CPS1
DIO1
BLTP1
SELE
ABCC8
TM6SF2
SI
MAT1A
FH
MB
SURF1
NQO1
GAA
ANGPTL8
BLTP3A
ACAD8
DHFR
PRL
LRP1
ASL
IVD
EDN1
TRMT10A
BCS1L
SLC19A1
MPO
PTPN1
HPRT1
OGDH
SLC37A4
LINC02605

NFE2L2
ICAM1
SLC16A1
BLTP3B
VEGFA
ATP7B
SEC14L3
ANO10
NAGLU
FBP1
VCAM1
AMPD1
AMPD3
POLG
SFTA3
BCKDHB
KCNJ11
SLC2A2
VKORC1
MC4R
ALPL
UNC119B
GSS
TFR2
CERT1
ABCC2
MIR27B
SDHA
APOH
TANGO2
ABCA4
FAH
MIR22
RFTN1
USH2A
AHCY
MIF4GD-DT
FAM3A
MIR140
HBB
IDUA
KL
SEC14L1
GAMT

ALDOB
NDUFS4
HSD17B13
PPIG
TTR
SERPINA1
MIR148A
SDHB
RYR1
ARG1
ILRUN
CYCS
GHR
MALAT1
SIRT6
ABCA12
GAPDH
ETHE1
PNPO
USF1
GH1
HAMP
GPIHBP1
H2AC18
TTPA
MIR125A
DDC
NPY
AGL
CCR6
DPAGT1
COX5A
TRA-TGC7-1
PDHX
NPPA
HSPG2
ATP5F1A
HJV
SEC14L4
TYMS
NR1D1
HPD
APBB1
PYGM

TFRC
APRT
PYY
SEC14L5
HLCS
PDK4
SLC5A2
PNPLA1
LRPPRC
MCCC1
SLC12A3
ADK
GNAS
PTS
CYP4V2
GLUL
CTH
ABCG2
BLTP2
GAS5
CD4
FABP12
KHK
ALG1
IGF2R
SEC14L6
MGAM
AR
FADS3
CD1A
CALCA
BMP6
TRIB1
MIR146B
NAGS
HP
IGFBP1
HLA-DRB1
IGFBP3
MCCC2
BHMT
GOT2
ACTB
GUSB

DOCK7
GLUD1
LEPQTL1
CLN3
PGR-AS1
LINC-ROR
ALDH4A1
FAM3B
TNFSF11
ECM1
VCP
TAT
MIR223
GLDC
ADIPOR1
MIR27A
SIRT3
DMGDH
CXCL8
FBN1
UGT1A
APOC4
BCKDHA
MIR483
SPP1
MIR144
SLC7A7
SCARB2
PDE5A
ST3GAL4
DDOST
XIST
FAS
LPXN
ALDH18A1
PDP1
MT-ND1
CA5A
MT-ATP8
DYRK1B
BDNF
MIR29A
RARRES2
VDAC1

ABCA2
TNFRSF11B
FAM3D
MYC
EPO
ALG8
PRKN
KCNQ1
SCARNA5
RRM2B
ELN
MT-CO1
H6PD
IAPP
DNM1L
PPP1R15B
COQ8A
ALG6
MIR199A1
APOM
FAM3C
FECH
MIR143
TCN2
STK11
PFKM
IGF2
SMARCA4
HYCC1
CLCNKB
FOXO1
ATP8B1
NDUFS2
COASY
CFH
PHKA2
ALPP
SLC34A1
OPA1
ANXA5
ABCC1
TLR2
TYMP
GALNS

CD1B
IFNG
BMAL1
MOCS2
CTSD
ZPR1
CIDEB
B2M
MIR23A
BCL2
GNPTAB
FOXC2
CYB5A
SLC40A1
ESR2
AOMS1
AHSG
MIR132
GATM
MIR30A
ALG2
NR1D2
THAP11
ATP7A
PANK2
VLDLR
HLA-B
CHROMR
NDUFB11
RAB7A
FOXO3
PVT1
SLC52A2
LCT
PHKB
SLC52A1
MIR145
SLC5A6
CACNA1S
CLPS
ATG2A
ODC1
EGFR
IL18

ATM
PEPD
GYS1
SLC12A1
MIR130A
EPRS1
STAT3
PRKAR1A
GNB3
FGFR4
SNORD15A
TMPRSS6
AGTR1
MIRLET7C
VPS13A
MT-RNR1
APOC4-APOC2
GALM
MIR192
DLEU2
FGFR1
GSK3B
PHKG2
OAT
DLG4
FTCD
SLC4A1
MSR1
TUG1
ATAD3A
MT-TE
SHMT2
GLP1R
COQ9
CYP4F12
MIR9-1
MIRLET7D
SHMT1
SLC11A2
APOD
PTPN11
CST3
BRAF
PGR

UMPS
FTH1
GLS
NR5A1
NDUFS1
UQCRFS1
SLC39A8
CELA2A
ERCC2
RPIA
MFN2
CD1D
TRC-GCA24-1
BLOC1S1
DBT
COX10
COSMOC
CUL3
MIA2
CAD
CAVIN1
AIFM1
MBP
GCLC
AASS
DNAH8
BRCA1
ABCA7
CD1C
SLC46A1
ZMPSTE24
AKR1C2
TOMM40
SERPINC1
GBE1
PHEX
PLIN4
NR0B2
SDHD
TH
MIR342
TPO
HSP90AA1
TTN

ALDOA
NAT1
DSP
BCAT2
MIR29C
STX1A
MYBPC3
MMP9
VWF
MIR142
KRT14
FTL
TRE-TTC3-1
SLC25A10
PDE3B
PRNP
DBH
SLC6A8
LBP
ALG13
SLC5A1
HTT
DYNC2LI1
CLU
HOGA1
ARG2
DNMT1
BSND
ATG7
FFAR4
COX4I1
GCH1
ISCU
CFHR1
SLC25A12
CYP2S1
PLAT
PARP1
APOL1
ERCC6
XK
COQ2
BMP1
CD44

MT-TK
VPS13C
CAV2
LRP5
MT-ND3
SLC25A15

Table S2. Primers used in the qRT–PCR analysis.

| Gene Name | Species | Sense Sequence (5' to 3') | Antisense Sequence (5' to 3) |
|---|---|---|---|
| Ces1d | Mouse | GGAGAGTCAGCAGGAGGTTTC | GAGGGACACACCACTCTCAG |
| Fasn | Mouse | TGGTGGTGTGGACATGGTCACAGA | CCGAAGCTGGGGGTCCATTGTGTG |
| Il10 | Mouse | CAAGGAGCATTTGAATTCCC | GGCCTTGTAGACACCTTGGTC |
| β-Actin | Mouse | ACTGCCGCATCCTCTTCCTC | AACCGCTCGTTGCCAATAGTG |
